# Determination of calcium and parathyroid hormone levels following hemithyroidectomy

**DOI:** 10.1186/s13044-021-00104-2

**Published:** 2021-06-03

**Authors:** Isabel Fernández Palop, Cristina Fernández Martínez, María Jesús Segura Giménez, M. Carmen Azorin Samper, Rafael García Fuster

**Affiliations:** 1 Universidad Católica de Valencia San Vicente Mártir, Valencia, Spain; 2 Hospital de Sagunto, Sagunt, Spain

**Keywords:** Hypocalcemia, Hemithyroidectomy, Intraoperative PTH

## Abstract

**Background and objective:**

Hypocalcemia is one of the main complications of thyroid surgery. We hypothesized that hemithyroidectomy may have an impact on serum parathyroid hormone (PTH) and calcium levels despite only one thyroid lobe is manipulated. The objective of this study was to analyze changes in serum PTH and calcium levels following hemithyroidectomy.

**Methods:**

This is a prospective study of 53 patients who underwent thyroid lobectomy. The serum PTH level was determined in the preoperative period, 15 min after extraction of the surgical specimen, and 24 h and 3 weeks after surgery. Serum ionized calcium was also measured in the preoperative period and at 6 h, 24 h and 3 weeks after surgery. We assessed the postoperative calcium value and its relationship with the extent of fall in PTH levels in the postoperative period.

**Results:**

None of the patients had the postoperative serum ionised calcium level less than 4 mg/dl. The decrease in postoperative calcium was statistically significant at 6 and 24 h after surgery; there was no difference at 3 weeks post-surgery. The change in post-operative serum PTH levels followed a similar trend to postoperative serum calcium levels.

**Conclusions:**

Although serum calcium level decreased after a lobectomy, it always remained above 4 mg/dl. We conclude that hypocalcaemia is rare following hemithyroidectomy.

## Background

Thyroid lobectomy or hemithyroidectomy is a surgical procedure that consists of the removal of half of the thyroid gland, preserving the contralateral lobe. Indications for this procedure include: unilateral symptomatic goiter, toxic adenoma or thyroid nodules with suspicious or undetermined characteristics in fine needle aspiration cytology [1].

The advantage of this technique is that it retains functional thyroid tissue, thus theoretically avoiding levothyroxine replacement therapy. In addition, it also reduces the risk of complications in the contralateral lobe.

One of the most frequent complications of thyroid surgery is post-surgical hypoparathyroidism and associated hypocalcemia.

Parathyroid hormone (PTH) is one of the three main hormones that modulate calcium and phosphate homeostasis. The other two are: calcitriol (1,25-dihydroxyvitamin D) and fibroblast growth factor 23 (FGF23) [2]. Control of serum ionized calcium concentration is mediated exclusively by PTH, maintaining its concentration within a narrow range by stimulating renal tubular and bone resorption [3, 4].

PTH also stimulates the conversion of calcidiol (25-hydroxyvitamin D) into calcitriol in renal tubular cells, thus stimulating intestinal absorption of calcium and bone turnover. Calcitriol feeds back to inhibit PTH secretion and inhibits PTH biosynthesis and proliferation of parathyroid cells.

When there is surgical injury to the parathyroid glands, insufficient PTH secretion occurs and, consequently, hypocalcemia develops. Severe hypocalcemia may produce symptoms such as seizures, cardiac arrythmias, heart failure or laryngospasm.

Although post-thyroidectomy hypocalcaemia is well documented in the literature, its incidence rate varies widely from 0.3 to 60% in different series [5–7].

Hypocalcemia due to hypoparathyroidism occurs, in most cases, without documented removal of the parathyroid glands. This fact implies that these glands are highly sensitive to the injury caused by surgical manipulation (ischemia, venous congestion). In the case of hemithyroidectomy, we assume that there will be no change in the serum calcium level when only one thyroid lobe is surgically manipulated, but there are few studies describing this.

In some studies, a higher incidence of hypoparathyroidism was found after total thyroidectomies compared to lobectomies or subtotal thyroidectomies [8, 9].

This study is based on the hypothesis that the manipulation of one of the two thyroid lobes can have some repercussion in the release of PTH and as a consequence serum calcium level.

The objective of our study was to compare the concentrations of serum PTH and calcium before and after hemithyroidectomy and to evaluate if there is a decrease in their concentrations in the post-operative period.

We also aimed to evaluate possible correlations between serum PTH and calcium levels.

## Material and methods

### Patients and procedures

This is a prospective observational study of patients who underwent hemithyroidectomy at our center between February 2016 and March 2019. The study was approved by the Center’s Ethics Committee. Patients with coexisting parathyroid or renal pathology were excluded.

A consecutive sampling was carried out, collecting data on the demographic characteristics of the patients and their thyroid pathology, the features of the surgery, the biochemical parameters and their evolution.

The following variables were recorded: sex, preoperative diagnosis, thyroid function, definitive pathological diagnosis, symptoms and signs of hypocalcemia, and other complications (recurrent laryngeal nerve paralysis, seroma, hematoma).

For the parathyroid glands, we recorded their number, condition and appearance at the end of surgery.

### Laboratory methods

A blood sample was taken before the operation to determine the basal levels of serum ionized calcium, phosphorus and intact PTH.

We performed ionized calcium measurements because some studies have shown these to be more reliable than total calcium measurements for immediate and long-term follow-up of patients after thyroidectomy [10].

An intraoperative PTH measurement was performed immediately after thyroid lobectomy (15 min after the surgical specimen was removed). The percentage decrease in post-operative PTH compared to the preoperative value was calculated as:

$$ \frac{\left(\mathrm{PTHpostop}-\mathrm{PTHpreop}\right)\ \mathrm{x}\ 100}{\mathrm{PTHpreop}} $$

A post-operative PTH level was determined 24 h and 3 weeks after surgery.

Ionized calcium and phosphorus measurements were obtained at 6 h, 24 h and 3 weeks after surgery.

Ionized calcium below 4.4 mg/dl was considered hypocalcemia. In patients with symptomatic hypocalcemia or with ionized calcium values equal to or less than 4 mg/dl, intravenous calcium, oral calcium, oral vitamin D, or a combination was administered.

Ionized calcium concentration was analysed with direct potentiometry with a calcium ion-selective electrode combined with an external reference electrode, in Radiometer ABL-835 gasometer. The ionized calcium value was corrected to physiological pH of 7.4.

Intact PTH was determined with the Cobas E801 analyzer from Roche Diagnostics by electrochemical luminescence enzyme immunoassay (EQL) analysis.

The laboratory reference ranges for biochemical parameters used in the study were: serum PTH (15-75 pg/ml), ionized calcium (4.40–5.20 mg/dl) and serum phosphate (2.5–4.5 mg/dl).

### Statistical analysis

Statistical analysis was performed using SPSS version 22 for Windows (Chicago, IL, USA).

We use the Student’s t-test to compare the pre and post thyroidectomy variables, and a *P*-value < 0.05 was taken as statistically significant.

## Results

A total of fifty-three hemithyroidectomies were performed in our center between February 2016 and March 2019. These were operated on by the same team of surgeons.

The centre is a secondary-level hospital, with 30 years of experience in thyroid surgery, where about 60 thyroidectomies are carried out every year.

### Patient characteristics

Demographic and clinical features of patients included in the study are shown in Table 1. There were more women (71.7%) then men (28.3%). The average age of the patients was 52.8 years (SD = 14.3).

**Table 1:** Patients characteristics

Characteristics	n (%)
**Total patients**	53
Male	15 (28.3)
Female	38 (71.7)
**Surgical indication**	
Bethesda category IV	23 (43.4)
Nodule size increase	20 (37.7)
Compressive symptoms	6 (11.3)
Hyperfunction	2 (3.8)
Recurrent cyst	2 (3.8)
**Lobectomy**	
Right	25 (47.2)
Left	28 (52.8)
**Definitive anatomopathological diagnosis**	
Hyperplasia	25 (47.2)
Follicular adenoma	20 (37.7)
Hashimoto's thyroiditis	4 (7.5)
Thyroid carcinoma	4 (7.5)
**Average age patients**	52.8 years (SD=14.3)

Similar percentages of the patients underwent a left and a right hemithyroidectomy.

The most common indications for surgery were: follicular proliferation (Bethesda category IV) (43.3%), increase in nodule size (37.7%) and compressive symptoms (11.3%).

The definitive anatomopathological diagnosis included hyperplasia (47.2%), follicular adenoma (37.7%), Hashimoto’s thyroiditis (7.5%) and thyroid carcinoma (7.5%).

The carcinomas found were all papillary and less than 5 mm in size. In all cases, the finding of malignancy was incidental.

Paralysis of the recurrent laryngeal nerve occurred in one of the patients after surgery. This paralysis was transient and corrected in less than 3 months.

### PTH and calcium levels

Preoperative mean serum calcium level was 4.8 mg/dl (SD = 0.15); at 6 h the mean was 4.52 mg/dl (SD = 0.2); at 24 h: 4.5 mg/dl (SD = 0.2) and at 3 weeks: 4.8 mg/dl (SD = 0.15).

The mean serum phosphate level was: 3.35 mg/dl preoperative (SD = 0.45); 3.38 mg/dl at 6 h (SD = 0.6); 3.10 mg/dl at 24 h (SD = 0.45) and 3.4 mg/dl at 3 weeks (SD = 0,5).

PTH decrease was statistically significant between preoperative measurement (mean: 53.84 pg/ml), and at 15 min (mean: 45.33 pg/ml, *p* = 0.04) and the day after surgery (mean: 42 pg/ml, *p* = 0.038). There was no difference between preoperative PTH and 3 weeks later (mean: 50.33 pg/ml).

**Fig. 1:**
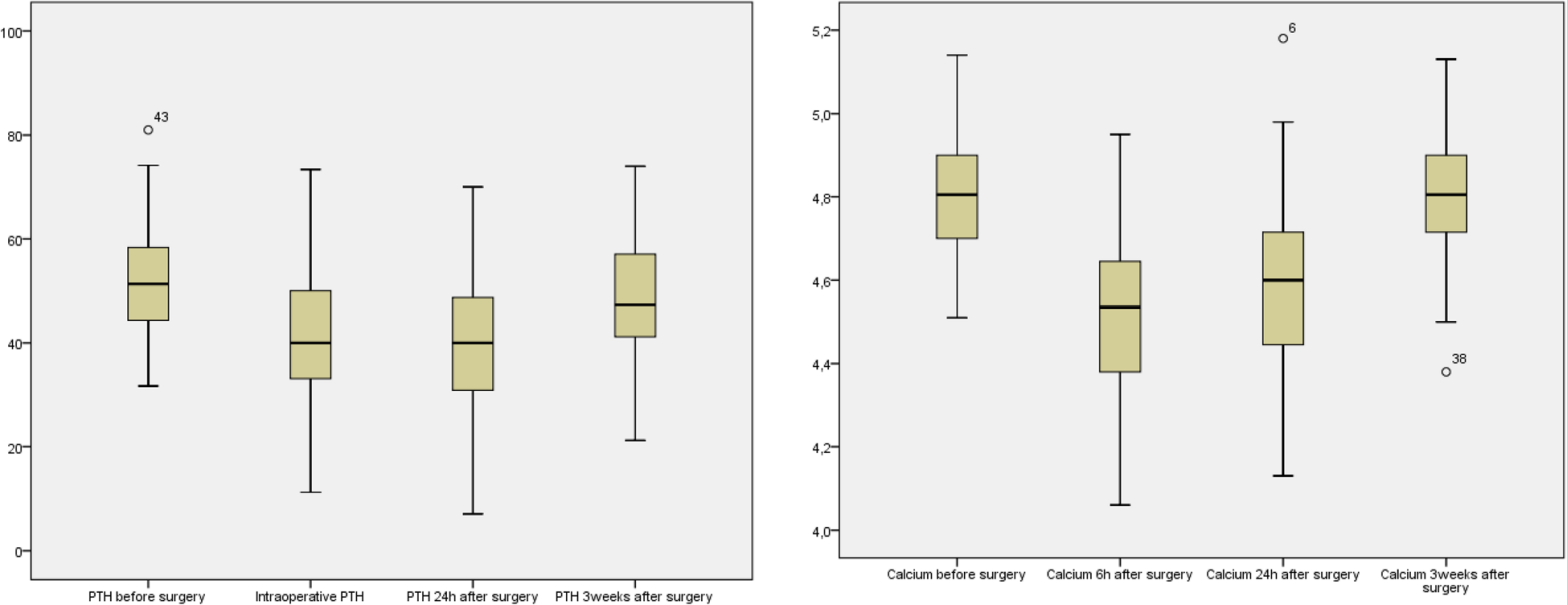
Longitudinal profiles in calcium and PTH

The longitudinal calcium and PTH profiles are shown in Fig. 1.

We have not been able to find any correlation between intra-operative PTH levels and calcium levels 6 h after surgery.

**Fig. 2:**
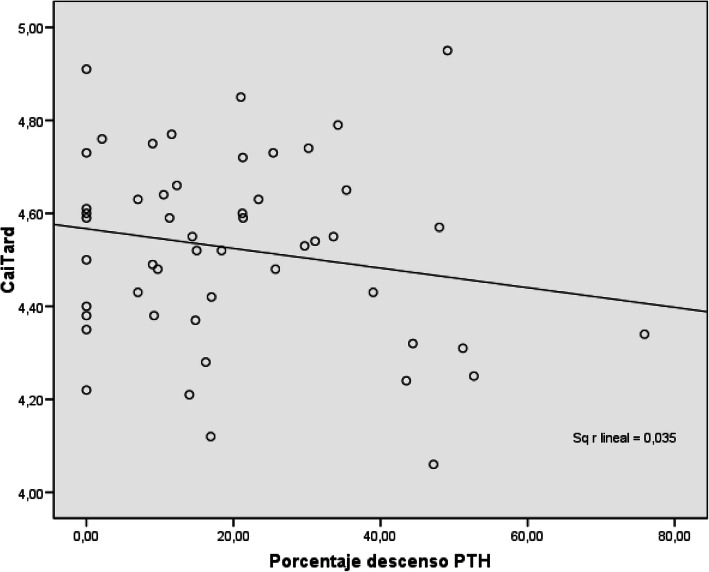
Pearson’s correlation for percentage decrease in PTH and calcemia obtained 6 h after the intervention

We compared the percentage decrease in PTH with the ionized calcium values at 6 h after surgery, and found an analogous trend. The trend line is descending, although R2 was not statistically significant (Fig. 2).

### PTH and calcium levels in relation to identification of parathyroid glands during surgery

The total number of exposed parathyroid glands was 106.

Two parathyroid glands were identified during the procedure in 21 patients (39.6%); a single gland in 28 patients (45.3%) and no parathyroid gland was identified in 4 patients (7.5%).

**Fig. 3:**
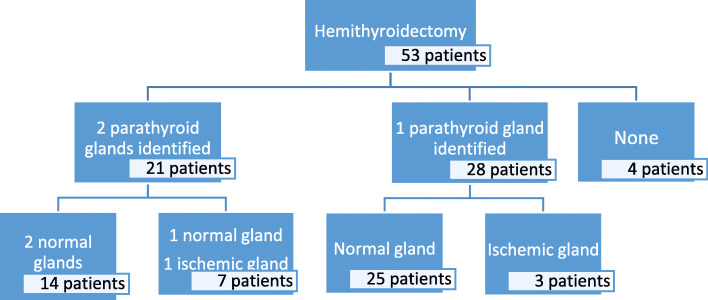
Parathyroid glands identified in surgery and their characteristics

The characteristics of the parathyroid glands observed in the three groups are shown in Fig. 3.

In two of the cases, the presence of a parathyroid gland was described in the histology specimen.

We analysed calcium and PTH levels in each of these groups.

In the group in which the two parathyroid glands identified as normal, the average serum calcium level 24 h after surgery is 4.56 mg/dl (SD 0.2) and the PTH 40.67 pg/ml. In the group in which one of the parathyroids was described as ischemic or congestive, the mean serum calcium level 24 h after surgery was 4.46 mg/dl (SD 0.2) and the PTH 48 pg/ml. In the group in which one parathyroid gland was found to in histology specimen the mean serum calcium level 24 h after surgery was 4.33 mg/dl (SD 0.3) and the PTH 47.7 pg/ml.

We observed that the mean serum calcium level after hemithyroidectomy is higher in patients in whom both parathyroid glands have been identified as normal at surgery as compared to the other two groups, although the difference was not statistically significant. We observed similar PTH levels among the three groups.

## Discussion

Hypocalcemia post-thyroidectomy as a multifactorial phenomenon is the most frequent clinical problem in patients undergoing thyroid surgery. The risk factors for post-thyroidectomy hypocalcaemia are: extension of the resection, group IV lymph node dissection, thyroidectomy as treatment of hyperthyroidism, central ligation of the lower thyroid artery, number of parathyroids identified and preserved during operation, and the surgeon’s experience [11].

In our study, the differences between preoperative and 6- and 24-h serum calcium levels reached statistical significance.

This confirms our hypothesis that manipulation in a thyroid lobe has an impact on postoperative ionized calcium concentrations, although this decrease is small and recovers before 3 weeks.

We found no significant difference between preoperative and three-week calcium. We also found no difference in serum calcium levels at 6-h and 24-h after surgery, so one of the two measurements would be sufficient to monitor calcium levels in patient undergoing hemithyroidectomy.

We found that the early postoperative PTH decrease was statistically significant even though PTH levels remained within the reference range. This fact confirms the impact of surgery on the parathyroid glands. This repercussion can be documented immediately.

We found no difference between preoperative PTH and the level 3 weeks later, since the two contralateral glands remain intact following hemithyroidectomy.

The fact that we found no correlation between postoperative PTH levels and postoperative calcium levels may be related to the fact that there was no postoperative hypocalcemia in the sample. However, we have found that the decrease in postoperative PTH follows a trend analogous to calcium levels.

Hemithyroidectomy may be an ideal procedure to perform in a Major Outpatient Surgery (surgical procedures of medium complexity for which the patient is admitted to the hospital on the day of the operation and is allowed to return home the same day).

The most serious complications (bleeding and recurrent laryngeal nerve paralysis) appear in the first hours after surgery [12, 13]. In the case of thyroid lobectomy, the possible recurrent laryngeal nerve paralysis would be unilateral, causing dysphonia, but not severe problems such as dyspnea or respiratory failure.

Our work allows us to guarantee the safety of the ambulatory surgery for hemithyroidectomy with respect to possible hypocalcemia. On the one hand, the decrease in serum calcium level does not reach below the normal reference range and, on the other hand, we can predict serum calcium level at 24 h post-surgery by measuring calcium level only at 6 h post-surgery.

## Conclusion

This study indicates that, in patients undergoing hemithyroidectomy, there are no significant differences between preoperative and postoperative serum calcium levels. Hemithyroidectomy is a safe intervention, in which post-operative hypocalcaemia below 4 mg/dl is rare.

The absence of differences between serum calcium levels at six and 24 h after hemithyroidectomy in this study suggests that if the serum calcium is normal at 6 h postoperatively, hypocalcemia will be unlikely in the subsequent hours. This fact allows us to perform lobectomy in outpatient surgery.

## Data Availability

The datasets used and analysed in the current study are available from the corresponding author on reasonable request.

## Abbreviations

PTH: Parathyroid hormone
